# Removing the Interdependency between Horizontal and Vertical Eye-Movement Components in Electrooculograms

**DOI:** 10.3390/s16020227

**Published:** 2016-02-14

**Authors:** Won-Du Chang, Ho-Seung Cha, Chang-Hwan Im

**Affiliations:** Department of Biomedical Engineering, Hanyang University, Seoul 04763, Korea; cross1279@hanyang.ac.kr (W.-D.C.); chayojs@naver.com (H.-S.C.)

**Keywords:** electrooculogram (EOG), bio-signal processing, EOG calibration, eye tracking

## Abstract

This paper introduces a method to remove the unwanted interdependency between vertical and horizontal eye-movement components in electrooculograms (EOGs). EOGs have been widely used to estimate eye movements without a camera in a variety of human-computer interaction (HCI) applications using pairs of electrodes generally attached either above and below the eye (vertical EOG) or to the left and right of the eyes (horizontal EOG). It has been well documented that the vertical EOG component has less stability than the horizontal EOG one, making accurate estimation of the vertical location of the eyes difficult. To address this issue, an experiment was designed in which ten subjects participated. Visual inspection of the recorded EOG signals showed that the vertical EOG component is highly influenced by horizontal eye movements, whereas the horizontal EOG is rarely affected by vertical eye movements. Moreover, the results showed that this interdependency could be effectively removed by introducing an individual constant value. It is therefore expected that the proposed method can enhance the overall performance of practical EOG-based eye-tracking systems.

## 1. Introduction

An electrooculogram (EOG) is the electric potential measured around the eyes, which is generated by the corneo-retinal standing potential between the front and back of the eye [1]. In recent decades, EOGs have been widely used to measure eye movement without a camera [2,3,4,5]. To measure eye movement, pairs of electrodes are generally attached either to the left and right of the eyes (horizontal EOG component) or above and below the eye (vertical EOG component) [2]. The horizontal and vertical EOG components are then obtained by subtracting the signal obtained at one electrode from the signal at the other electrode [2,3,4]. This bipolar measurement method has been widely utilized in various applications since 1936, when it was first revealed that EOG components reflect the horizontal and vertical movements of eyes [5].

To date, many studies have strived to recognize user intention using both the horizontal and vertical EOG components; however, most of these studies have been limited to only the estimation of momentary eye movements. The most successful application of EOG was as a substitute for the standard four directional (arrow) keys. For example, EOG-based gaze-direction estimation has been applied to simple eyeball input devices [6], a wheelchair controller [3], and a computer game controller [7]. Only a few studies have attempted to classify more than four eye-gaze directions. Bulling *et al.* [2] sought to recognize the daily activities of users, such as reading books and watching videos, by estimating 16 eye-gaze directions from EOG data. However, the individual classification accuracy was not reported. The most advanced study was recently performed by Yan *et al.* [8]. It classified 24 directions with fairly high accuracy, although the number of subjects tested was only three. Although a number of studies have estimated single momentary eye movements from EOG, studies to continuously track eye movements and estimate eye-gaze patterns have rarely been performed, and the accuracy has generally been limited. Tsai *et al.* attempted to recognize ten eye-written digits and four extra symbols from 11 subjects. The overall accuracy was reported to be 72.1% [9].

Many possible reasons exist for the limited performance of EOG-based eye tracking. Muscular artifacts, eye blink artifacts, and brain signals (electroencephalogram (EEG)) are the major artifacts contaminating EOG [10]. Moreover, ambient light conditions and the subject’s state of alertness can additionally affect the EOG signals [11]. Studies in the last few decades on this issue have sought to improve the estimation accuracy by adopting specific signal processing techniques, such as digital filters and wavelet transforms [2,12]. For example, Joyce *et al.* [10] estimated the propagation matrix between recorded signals and intended eye movement. Nevertheless, it has been reported that the vertical EOG component has lower stability than horizontal EOG, thereby making accurate estimation of the vertical location of the eyes difficult [10].

The main objective of the present work is to introduce a method to eliminate the undesired interdependency between horizontal and vertical EOG components. To the best of our knowledge, this objective has not been considered in previous studies to date. Through experimental studies, we investigate whether the proposed method can enhance the performance of EOG-based eye tracking.

## 2. Experimental Environment

### 2.1. Recording of EOG Signals

**Figure 1:**
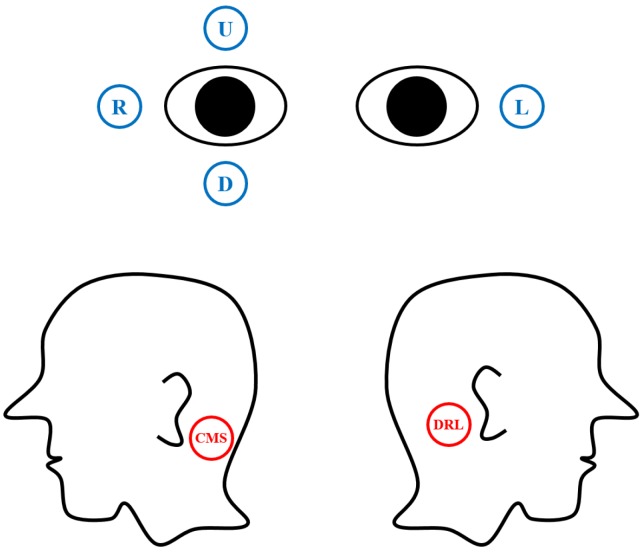
Electrode placements used to record the horizontal and vertical EOG components.

EOG signals were recorded at a sampling rate of 2048 Hz by using an ActiveTwo biosignal recording system (BioSemi, Amsterdam, The Netherlands) with six flat-type active electrodes. Two pairs of electrodes were placed at the most widely used locations to record EOG: to the left and right of the eyes, and above and below the right eye. Reference and ground electrodes were attached at left and right mastoids. Details of the electrode configurations are illustrated in Figure 1. The horizontal EOG component was obtained by subtracting L from R, and the vertical EOG component was obtained by subtracting D from U, where L, R, D, and U represent the electric potential recorded from the corresponding electrodes in the figure. Two electrodes—common mode sense (CMS) and driven right leg (DRL), which operate as the reference and ground—were placed at the left and right mastoids, respectively [13].

When placing the electrodes on the skin, a conductive gel was applied on the electrodes together with double-sided adhesive disks. This gel reduces variations of the skin-electrode capacitance due to the irregular surface of the skin and thereby enables more reliable bioelectric signal measurements. We performed no skin preparation processes (e.g., cleaning skin with alcohol) before attaching the EOG electrodes.

### 2.2. Experimental Setups

**Figure 2:**
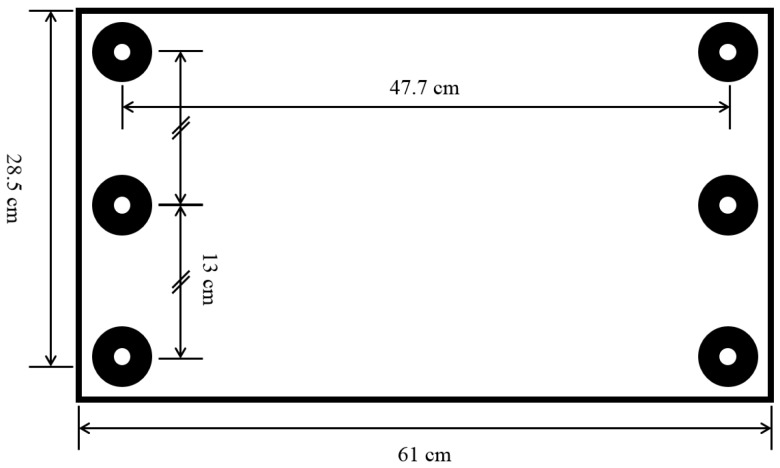
Size of the monitor and six dots arranged in a 3 × 2 grid. The six dots were used as targets for drawing patterns.

EOG signals were acquired from ten participants (between 18 and 25 years of age). Prior to the data acquisition, a comprehensive summary of experimental procedures and protocols were explained to each participant. All participants signed a consent form and received monetary reimbursement for their participation. The participants were situated a distance away from a monitor in a quiet room. The distance from the eyes to the monitor was set to 62.5 cm in our experiments. To exclude the potential influence of head movement, the head of each participant was fixed using a type of chin rest generally used in ophthalmic applications [14]. The height of the chin rest could be adjusted for the convenience of the participants. On the monitor facing the participants, six dots were arranged on a 3 × 2 grid. The size of the monitor was 28.5 cm × 61 cm (height × width). The distance between the left and right dots was 47.7 cm, and the distance between the two vertically adjacent dots was 13 cm. The arrangements of the six target dots are depicted in Figure 2.

The dots were displayed via E-Prime (Psychology Software Tools, Inc., Sharpsburg, PA, USA), which is a software package for synchronizing various events with biosignal recording systems via a parallel port. 

**Figure 3:**
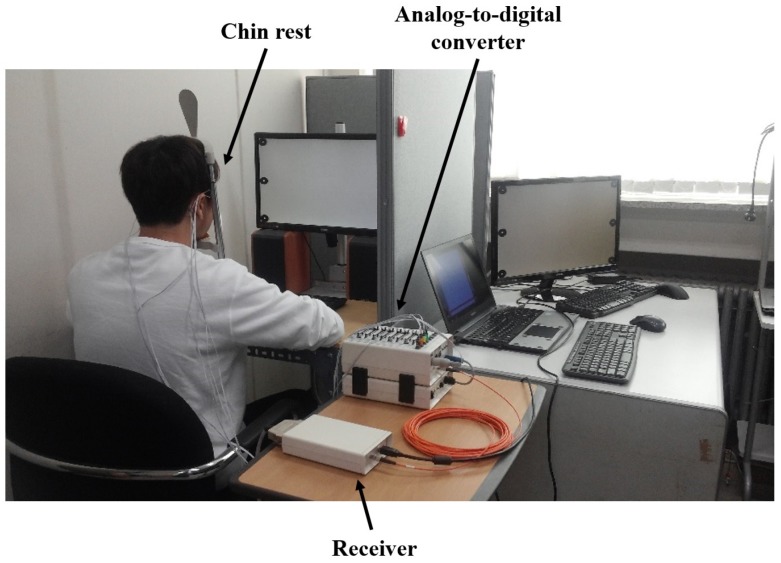
Snapshot of overall experiment environment. A chin rest was used to prevent potential head movements. The EOG data, collected by ActiveTwo, were transferred to a laptop for recording. Another computer (under the desk) was used to display dots and instructions to each participant and synchronize events with the recorded data.

E-Prime was used to visualize graphical instructions and target dots on the monitor, as well as to synchronize the experimental paradigm with the recording device by transmitting trigger signals to the system (receiver). A snapshot of the overall experimental environment is shown in Figure 3.

## 3. Methods

### 3.1. Preprocessing

For better estimation of eye movements from EOG, noises and artifacts were removed according to the general steps used for previous EOG-based eye movement estimation studies, which were high-frequency noise removal, baseline removal, eye blink removal, and saccadic detection [2,9,10]. The EOG signals were resampled at a sampling rate of 64 Hz and were median filtered to remove high-frequency noises. The width of the median filter window was empirically set to four. The median filter was employed for the high-frequency noise removal because it was known to preserve features of eye-movement signals well [2]. To remove signal baseline drifts, the median value of the baseline period (a 100-ms signal before drawing a target pattern) was subtracted from the signals being analyzed.

Eye blink artifacts are often included in EOG and must be detected in the preprocessing phase because they are easily misrecognized as vertical eye movements. An eye blink detection method [15] utilizing a digital filter—called the maximum summation of the first derivative in a sliding window (MSDW)—was employed for this purpose. This method can detect the extents of eye blink periods accurately from a single-channel signal without applying any machine learning algorithms (e.g., [16]). The MSDW filter is defined as:

(1) MSDW(t)=s(t)−s(t−K)

(2) $$K=\arg\underset{k}{\max}\left\{\left|s\left(t\right)-s\left(t-k\right)\right|\right\}$$

subject to M(t,t−k)=m(t,t−k) and $\text{min}\left\{s^{\prime }\left(t\right),s^{\prime }\left(t-k\right)\right\}\leq \forall s^{\prime }\left(t-o\right)\leq \text{max}\left\{s^{\prime }\left(t\right),s^{\prime }\left(t-k\right)\right\}$, where s(t) is a source signal at time *t*, $s^{\prime }\left(t\right)=s\left(t\right)-s\left(t-1\right)$, u≤k≤U, 1≤o≤k−1, and M(a,b) and m(a,b) respectively denote the number of local maxima and minima within a range [a,b]. Furthermore, [u,U] is the expected range of the slope width for eye-blink artifacts. When multiple K’s are found in Equation (2), the smallest K is selected to calculate the filter output; and u is chosen for K if any k does not satisfy the above conditions. The set of eye blink ranges is determined using the following equation:

(3) $$R=\left\{\left[\begin{array}{cc} T\left(Max_{i-j}\right)-\left|W_{MSDW\left(T\left(Max_{i-j}\right)\right)}\right|, & T\left(Min_{i}\right) \end{array}\right]\right\}$$

where $Max_{i}$ and $Min_{i}$ are the *i*th local maximum and minimum in the filtered signal, respectively, j is a positive integer that maximizes the $Max_{i-j}-Min_{i}$ subject to $Max_{i-j}-Min_{i}>\theta$, $Max_{i-j}>\theta \cdot \alpha$, $Min_{i}<-\theta \cdot \alpha$, $T\left(Max_{i-j}\right)-T\left(Min_{i}\right)\leq M$. In addition, θ is a threshold for determining eye blinking, α is a ratio to discard saccades, and $T\left(Max_{i}\right)$ and $T\left(Min_{i}\right)$ are the time points of the *i*th local maximum and minimum, respectively. Any j is rejected if the range related to j partially overlaps with another range. After all the ranges were determined, any range that is fully overlapped by another range is discarded. The data in the detected eye-blink regions are removed and then the missing data are replaced by linearly interpolated data. That is to say, a data point s(t) within the removed region is interpolated as:

(4) $$s\left(t\right)=s\left(a\right)+\frac{s\left(b\right)-s\left(a\right)}{b-a}\left(t-a\right)$$

To remove the unknown sources of artifacts and extract signals only related to saccadic eye movements, we adopted the continuous wavelet transform-saccade detection (CWT-SD) algorithm of Bulling *et al.* [2]. This algorithm determines signals in a range as the eye-movement signals if the absolute values of the wavelet coefficients of the signals are greater than a preset threshold. The wavelet coefficient $C_{b}^{a}$ of data *s* at scale *a* and position *b* is defined as:

(5) $$C_{b}^{a}\left(s\right)=\int_{-\infty }^{\infty }s\left(t\right)\frac{1}{\sqrt{a}}\bar{\psi \left(\frac{t-b}{a}\right)}dt$$

where ψ represents a Haar mother wavelet. The wavelet scale used to calculate the coefficients was set to 20, as suggested by Bulling *et al.*, and the threshold was empirically set to 0.08. The signals were normalized to have a width of one before the saccade detection in order to apply the same threshold.

### 3.2. Study on EOG Dynamics

**Figure 4:**
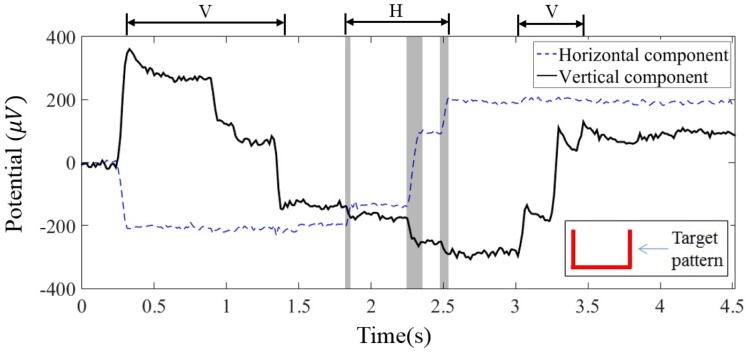
Horizontal and vertical EOG components of a participant. V and H above the graphs denote the time periods of the vertical and horizontal eye movements, respectively. The horizontal axis represents time.

In this section, we visually investigate the changes in the EOG signals recorded while the subjects moved their eyes to draw a specific pattern. EOG signals were recorded from ten participants, as described in Section 2.2. The recorded signals were resampled, and the noises and artifacts were removed as described in the previous section. Figure 4 shows vertical and horizontal EOG components recorded while a participant drew an angulate U shape starting from a dot on the top-left. The participant sequentially moved his/her eyes in three steps (vertically, horizontally, and vertically) according to the shape of the pattern. Consequently, the amplitude of the vertical EOG component was expected to decrease, be unvaried (flat), and increase at each step, if the component does not have any influence from the other signal source (Let us call the expected signal an “ideal” signal). The ideal amplitude of the horizontal component was expected to be unvaried, increase, and unvaried in a series.

As clearly seen from the example of the EOG components in Figure 4, the vertical component had downward phantom movements as the eyes moved from left to right, especially when the horizontal component drastically changed (gray regions in Figure 4). On the contrary, the horizontal EOG component was stable during the vertical eye movements (time periods of 0.3–1.4 s and 3–3.5 s, which were determined by a visual inspection of the recorded EOG signals). This apparent influence of the horizontal eye movement on the vertical EOG component makes accurate estimation of the vertical location of the eyes difficult. It can thus degrade the overall accuracy of EOG-based eye tracking.

**Figure 5:**
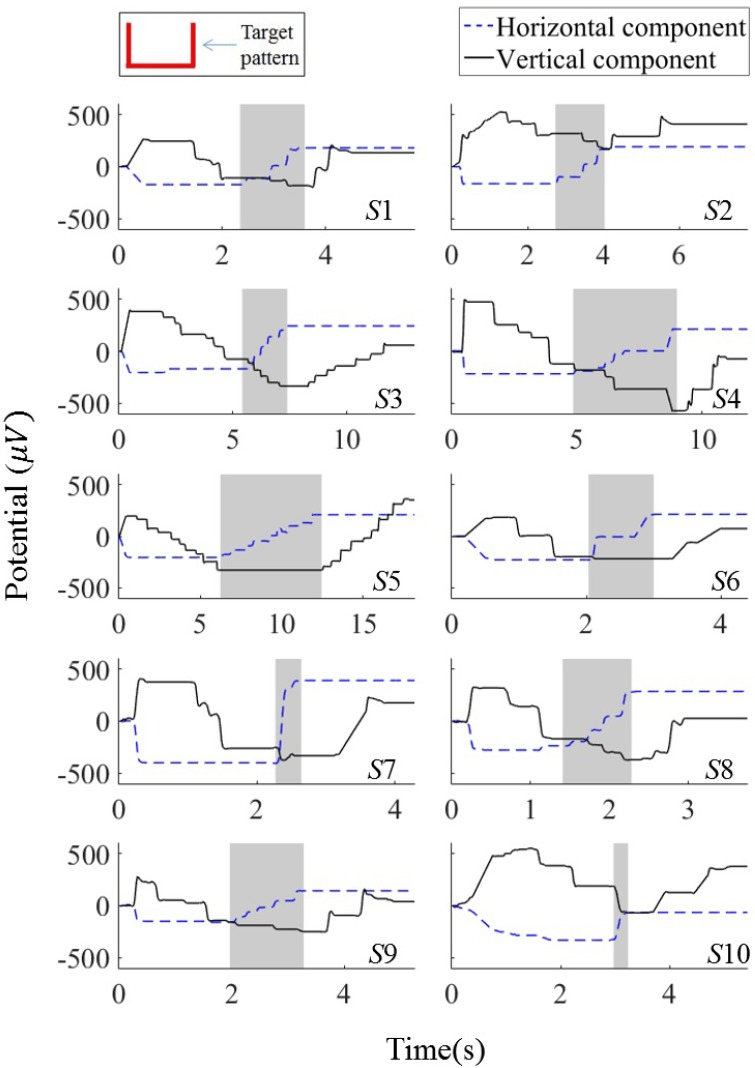
Horizontal and vertical EOG components for all subjects after saccade detection. The subject identification numbers are indicated at the bottom-right of each plot (from *S*1 to *S*10). The shaded regions indicate the time periods in which the subjects moved their gazes in the horizontal direction.

For further analysis of this interdependency, we visualized EOG components of all participants, as shown in Figure 5. In this figure, the horizontal components well follow the ideal signal trends, showing a gradual increase in the middle region, but minimal signal variations in other regions. The vertical components, however, significantly differ from the ideal signal. In most cases, it is observed that the amplitudes of the vertical EOG components decrease during the horizontal eye movements. This interdependency between the vertical and horizontal EOG components is observed for all subjects except one (Subject 5), although the degrees of the interdependency individually differed.

### 3.3. Removal of Interdependency between Horizontal and Vertical EOG Components

In the previous section, we confirmed that the vertical EOG components are highly influenced by horizontal eye movements, and that the degrees of the interdependency differ among individuals. This interdependency can be readily removed by estimating the individual degree of interdependency if it is assumed to be stable over time. Under this assumption, the vertical component is compensated by:

(6) $${\text{EOG}}_{v,\alpha }=\left(U–D\right)–\alpha \;{\text{EOG}}_{h}$$

where EOG*_h_* = *R* − *L* and *U*, *D*, *R*, and *L* denote the signals recorded from the corresponding electrodes shown in Figure 1. α is a constant determined for each individual using the EOG signals recorded during eye movement from left to right. Because the main objective of this compensation process is to stabilize the vertical component as much as possible during pure horizontal eye movement, α is determined to minimize the variance in the vertical component during pure horizontal eye movement. In other words:

(7) $$\alpha =\text{arg }\underset{k}{\min}\left\{\sigma ({\text{EOG}}_{v,k})\right\}$$

where σ denotes the standard deviation.

### 3.4. Experimental Validation of the Proposed Compensation Method

**Figure 6:**
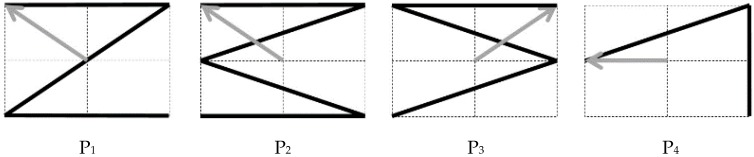
Four different eye-gaze patterns used for the experiment.

An experiment was designed to investigate the enhancement of EOG signals by the introduction of an individual constant given in Equation (7). For the validation of the proposed compensation method, we compared the original (uncompensated) EOG components with those obtained after the proposed compensation process, as well as the ideal (desired) EOG components. EOG signals were recorded from the ten participants introduced in Section 3.2 while they were visually drawing four different target patterns (denoted as P1–P4, as shown in Figure 6).

**Figure 7:**
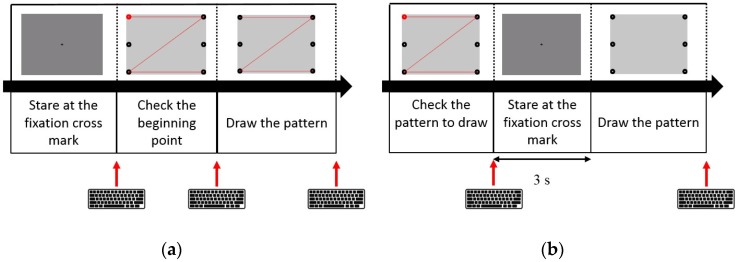
Schematic illustration of the experimental procedures: (**a**) Session to practice eye drawing of patterns; and (**b**) Session to record the EOG signals. The image of a keyboard implies that a user’s response is required to proceed to the next step.

In this experiment, the participants were provided with a short practice session to foster familiarity with the experiment before the data acquisition (EOG signals were not recorded during this session). The participants were asked to gaze at a fixation cross at the center of the monitor for 3 s. Then, a target pattern was displayed together with a red dot marking its starting point (see Figure 7a). When the participants recognized the shape of the target pattern and the location of the starting point, they began to draw the target pattern along a guide line after pressing a key in the keyboard in front of them. They were instructed to move their gaze at a constant speed along the path that appeared on the monitor. When they finished drawing the pattern, they pressed the key once again to proceed to finish drawing the pattern. They practiced drawing four patterns three times each.

The procedure of the data acquisition was similar to the practice session; however, the guide lines were not provided during the pattern drawing. Instead, the target pattern and starting point were displayed on the monitor for 3 s before the presentation of the fixation cross. After gazing at the fixation cross for 3 s, the participants started to draw the target pattern without guide lines, and they pressed a key to finish the eye drawing. The four target patterns were drawn three times for the nine subjects and twice for one subject. All events, such as pressing a key or changing pages, were also recorded and synchronized with EOG signals using E-Prime.

After the data acquisition and preprocessing procedures, the signals were resampled to obtain a similar Euclidean distance between adjacent data points. The resampling process is a recommended procedure [17] for character recognition because different writing speeds over participants may affect the precision of the similarity measure. Because the objective of this experiment was to compare the shape of the compensated signals and the ideal signals, this procedure was deemed necessary. The resampling procedure was as follows. We (1) selected the first point of the signal; (2) then selected a point during the point-by-point progression from the first to the end if its distance to the most recently selected point was larger than the criterion; and (3) added n−1 points and interpolated them when the distance was larger than *n* times that of the criterion points. We set m+σ as the criterion, where m and σ are the mean and standard deviation of the distance, respectively. The resampled signals were then compensated using the individually estimated degree of interdependency (α), where the degree value was individually calculated with the signal-trial EOG data given in Figure 5.

Next, the accuracy of the compensation was quantitatively evaluated by comparing the compensated signals with the ideal signals. For the quantitative comparison, the similarity between the two vertical components was evaluated using Pearson’s correlation coefficient together with dynamic time warping (DTW) [18]. DTW is often used to align corresponding points between two time-series signals. It can help obtain a more accurate estimate of similarity regardless of the length of the signal or drawing speed. The sampling of the ideal signal was set to have the same data points to the test signal for each comparison. This procedure guarantees that the length of a signal does not influence the comparison results. Details of DTW are described in Appendix A. After aligning the corresponding points using DTW, the correlation coefficient was calculated between two signals for the comparison. The horizontal component was not evaluated because the horizontal EOG component was not influenced by the vertical eye movements, as shown in Section 3.2.

## 4. Results

**Figure 8:**
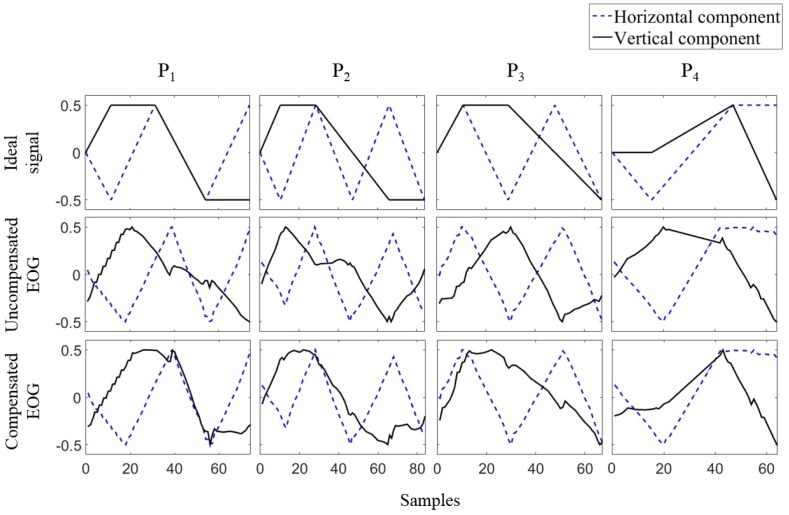
Examples of EOG signals acquired when a subject visually drew four different patterns, where the values were normalized to [−0.5, 0.5].

Figure 8 depicts examples of the horizontal and vertical EOG components acquired while Subject 10 visually drew patterns P1–P4 shown in Figure 6. The “ideal signal” represents ideal EOG components, assuming a constant eye-gaze speed. The “uncompensated EOG” represents the estimated gaze without considering the interdependency between the two EOG components. The “compensated EOG” represents the signal compensated by Equations (6) and (7). Figure 8 shows that the horizontal component is similar to the ideal waveform; however, the vertical component is severely distorted during the horizontal eye movement before applying a compensation method. It is evident that the compensated vertical EOG component corresponds much better with the ideal waveform than does the signal before compensation.

Table 1 lists the summary of the average correlation coefficients between the recorded (compensated and uncompensated) and ideal vertical EOG components. The correlation coefficients of the compensated EOG (average 0.95) is significantly higher than that of the uncompensated EOG (average 0.90) (paired *t*-test, $p<10^{-7}$).

**Table 1:** Pearson’s correlation coefficients between the recorded vertical EOG components and ideal EOG components.

Patterns	P1	P2	P3	P4	Average
Compensated EOG	0.97 ± 0.05	0.96 ± 0.08	0.98 ± 0.02	0.91 ± 0.09	0.95 ± 0.07
Uncompensated EOG	0.92 ± 0.08	0.93 ± 0.08	0.92 ± 0.06	0.79 ± 0.20	0.90 ± 0.13

Table 2 lists the individual compensation constant α and the corresponding correlation coefficients for each individual. The data in the table confirm that the interdependency levels vary across subjects, and this interdependency can be effectively removed by applying the compensation constant. The compensation constant varies from −0.48 to 0 (mean: −0.21, standard deviation: 0.18). In this result, six participants showed statistically significant increase in the correlation coefficient after the compensation process, but the other four participants (Subjects 5 and 7–9) showed smaller increases in the correlation coefficient. This table additionally demonstrates that the individual compensation constant *α* does not need to be updated for a given subject unless the electrode locations change. It would be noteworthy to examine in future studies the cause of these large individual variations in the compensation constant. Furthermore, this individual difference in the interdependency between two EOG components may be potentially used as a new feature for biometric verification [19], although confirmation of this notion requires further research.

**Table 2:** Individual compensation constants and Pearson’s correlation coefficients. The *p*-values were calculated between the coefficients of the vertical components of the compensated and measured EOGs. The *p* value for Subject 5 was not calculated because the two EOGs were identical.

Subject ID	α	Compensated EOG	Uncompensated EOG	*p*-Value
S01	−0.12	0.93 ± 0.05	0.90 ± 0.04	0.001
S02	−0.25	0.94 ± 0.07	0.81 ± 0.16	0.007
S03	−0.35	0.91 ± 0.13	0.80 ± 0.18	0.021
S04	−0.5	0.92 ± 0.12	0.79 ± 0.14	0.006
S05	0	0.98 ± 0.02	0.98 ± 0.02	-
S06	−0.02	0.97 ± 0.02	0.97 ± 0.02	0.022
S07	−0.04	0.97 ± 0.03	0.98 ± 0.01	0.181
S08	−0.22	0.96 ± 0.06	0.93 ± 0.03	0.066
S09	−0.13	0.98 ± 0.02	0.97 ± 0.02	0.099
S10	−0.48	0.97 ± 0.02	0.80 ± 0.18	0.005

## 5. Conclusions

In this study, we investigated the changes of vertical and horizontal EOG components during eye tracing of specific patterns. Experiments conducted with ten participants showed that the horizontal eye movement can influence the vertical EOG component, although the degrees of this interdependency showed large inter-individual variability. Therefore, we proposed a method to eliminate this unwanted interdependency between horizontal and vertical EOG components by introducing an individual constant, which can be readily obtained from a short period of EOG signals recorded during a single “left-to-right” movement. The experimental results showed increases in the correlation coefficient with ideal EOG waveforms ($p<10^{-7}$), demonstrating that the EOG signals could be significantly enhanced by using the proposed compensation method. It is expected that the proposed method can be utilized for a variety of applications of human–computer interaction (HCI), such as EOG-based wheelchair controller, a new type of game input devices, and eye-writing systems. In addition, one of the possible applications of these eye movement-based HCI systems is a communication platform for patients with amyotrophic lateral sclerosis, generally known as Lou Gehrig’s disease. Since these systems generally require reliable and accurate estimation of eye-movement, the proposed method would be an easy and effective way to enhance the system performance. In the future studies, we intend to further investigate the stability of the individual constant value during a longer term period, of which the results might be used to determine a required frequency of EOG recalibration.

## Appendix A

This research uses a method called dynamic positional warping to align two signals. This method is a type of dynamic time warping (DTW) algorithm. The alignment is determined to minimize the dissimilarity between two signals, *A* and *B*, where the dissimilarity is defined as follows:

(A1) $$d_{dtw}=\tau \left(L_{A},L_{B}\right)$$

(A2) $$\tau \left(i,j\right)=\left|\left\{A\left(i\right)-A\left(i_{prev}\right)\right\}-\left\{B\left(j\right)-B\left(j_{prev}\right)\right\}\right|+\omega \left(i,j\right)$$

(A3) $$i_{prev}=i-C_{A}\left(c_{\text{min}}\left(i,j\right)\right)$$

(A4) $$j_{prev}=j-C_{B}\left(c_{min}\left(i,j\right)\right)$$

(A5) $$c_{min}\left(i,j\right)=\underset{c}{\text{argmin}}\left\{\tau \left(i-C_{A}\left(c\right),j-C_{B}\left(c\right)\right)\right\}$$

(A6) $$\omega \left(i,j\right)=\underset{c}{\min}\left\{\tau \left(i-C_{A}\left(c\right),j-C_{B}\left(c\right)\right)\right\}$$

where $L_{A}$ and $L_{B}$ are the lengths of ideal and test signals, respectively, τ(1,1) = 0, $C_{A}\left(c\right)$ and $C_{B}\left(c\right)$ are the *c*th slope constraints on the pattern axes, which limit the number of skipping data points. The pair of the constraints ($C_{A,B}$) can be written as:

(A7) $$C_{A,B}=\left\{\left(1,m\right),\left(m,1\right)|1\leq m\leq M\right\}$$

where *M* is the maximum branch length of slope constraints.
